# Enzymatic Digestion of Calf Fleshing Meat By-Products: Antioxidant and Anti-Tyrosinase Activity of Protein Hydrolysates, and Identification of Fatty Acids

**DOI:** 10.3390/foods10040755

**Published:** 2021-04-02

**Authors:** Tullia Tedeschi, Cecilia Anzani, Maura Ferri, Silvia Marzocchi, Maria Fiorenza Caboni, Stefania Monari, Annalisa Tassoni

**Affiliations:** 1 Department of Food and Drug, University of Parma, Parco Area Delle Scienze 49/a, 43124 Parma, Italy; anzani.cecilia@gmail.com; 2 Department of Biological, Geological, Environmental Science, University of Bologna, Via Irnerio 42, 40126 Bologna, Italy; maura.ferri@unibo.it (M.F.); stefania.monari2@unibo.it (S.M.); 3 Department of Civil, Chemical, Environmental and Materials Engineering, University of Bologna, Via Terracini 28, 40131 Bologna, Italy; 4 Department of Agricultural and Food Sciences and Technologies, University of Bologna, Piazza Goidanich 60, 47521 Cesena, Italy; silvia.marzocchi4@unibo.it (S.M.); maria.caboni@unibo.it (M.F.C.)

**Keywords:** by-products valorization, protease, bioactive peptides, protein hydrolysates, fatty acids

## Abstract

The food waste reduction through an efficient recovery of its valuable building molecules has become an important topic with a positive effect on the economy and the environment. In this work, the revalorization of slaughterhouse calf fleshing meat through its enzymatic hydrolysis is proposed. The proteolytic activity of 11 enzymes was initially screened and the four most efficient enzymes (papain, trypsin, pancreatin, and bromelain) were selected. The molecular profiling of the different protein/peptide fractions by the Linear Trap Quadrupole-OrbiTrap technique showed compositional differences due to the specificity of the enzymes’ cleavage sites. In order to find a potential reuse of these hydrolysates, the analysis of antioxidant and, for the first time on fleshing meat hydrolysates, of anti-tyrosinase activities, was performed. Papain-digested samples were those showing the highest inhibition activity of tyrosinase enzyme (55.6%) as well as the highest antioxidant activity (3.52 g TEAC/L). In addition, the composition analysis of the lipid fraction was performed. The mono-unsaturated fatty acids resulted to be the most abundant lipid in all the samples with the exception of pancreatin-treated hydrolysates in which poly-unsaturated fatty acids were predominant. The present results seemed to support a possible valorization of isolated fractions from calf fleshing by-products, as food or feed ingredients, by the implementation of fraction isolation within the meat-processing pipeline.

## 1. Introduction

The recovery of food waste and by-products has become a hot topic, because of the rising global demand of animal protein-based food products up to 70% by 2050 [1]. This fact is due to the population growth and to the adoption, in underdeveloped countries, of Westernized eating habits. Furthermore, this expected increase of animal protein product consumption leads to a simultaneous increase of the production of food-related waste [2]. Interestingly, depending on animal species, after the slaughtering, only 30–40% of meat-processing products is used for human consumption [3]. Particularly in the case of beef, that 44% is considered as “meat” and the remaining 56%, usually discarded, is composed of non-edible (e.g., specified risked materials, SRM) and edible parts (e.g., offal) [4]. The incorrect utilization or the non-utilization of these by-products causes loss of potential revenues and rising disposal costs for the producing companies [5]. An efficient and sustainable utilization of these by-products will have a positive economic and environmental impact. Moreover, these by-products could be exploited as a cheap source of valuable compounds, which could be recovered and recycled as functional additives or ingredients in different food or feed products [6].

Beef fleshing meat by-product is represented by the meat portion, which is directly attached to the bovine hides. This part is usually discarded and not recovered despite its potential as a valuable source of proteins. Different strategies have been proposed [7] for the recovery of fleshing meat. Previous studies reported the use of the enzymatic hydrolysis on fleshing meat from different animal species [8,9,10,11] with or without the chemical treatments of tanning process. More recently, the activity of six proteolytic enzymes was compared, and the protein fraction composition characterised [12]. The meat by-product hydrolysates could find valuable industrial applications in the food sector as they constitute a notable source of nutrients like amino acids, minerals, and vitamins, they can be used to improve technological functions (flavouring compounds, water binding agents, emulsifiers), enhance sensory qualities (colour, texture, flavour), and increase the shelf life [13]. Moreover, a prevalent fraction of the meat hydrolysates is constituted by bioactive peptides, which could be used for the production of functional ingredients. Bioactive peptides are defined as a short sequence of amino acids, which are not active within the structure of the parent protein but show a positive effect on the system of the body once released [14]. During the proteolysis, a high number of peptides are liberated, but only a few of them can exhibit determined health benefits and can be defined as bioactive peptides [15]. Some examples of bioactivities, which can affect the health of the consumers, are anti-hypertensive, antioxidant, anti-inflammatory, antimicrobial, anticancer, anti-thrombic, and opioid-like [14]. Oxidation in food is directly linked to its deterioration and, in particular, it affects lipids, carbohydrates, and proteins. In meat products, the most important effects of deterioration are colour changes, microbial growth, and lipid oxidation [16]. To prevent the oxidation in food, synthetic antioxidants are usually added to retard lipid oxidation [17] but their utilization is under strict regulation as they might be associated with health risk. On the other hand, enzymatic browning due to tyrosinase activity may further result in discolouration and reducing the nutritional value of foods [18]. Therefore, nowadays, industries are searching for tyrosinase inhibitors to be used as browning preventing agents in nutraceutical and functional food sectors [19] or as anti-aging ingredients in cosmetic field [20]. This biological activity was usually ascribed to plant secondary metabolites [18,21,22] and, recently, also to proteins and peptides coming from both animals and plants [23,24,25]. Anti-tyrosinase capacity of fleshing meat hydrolysates is reported here for the first time and could prove to have good possibilities of future exploitation already in the meat processing industries. In fact, the protein hydrolysates coming from meat by-products and showing anti-browning capacity could be valuable, both as preservatives for meat-based food products, as well as additives for active food packaging.

As regards fatty acid total content, saturated fatty acids are the preponderant in bovine meat. Because of the hydrogenation process of unsaturated fatty acids that make them saturated that occurs in bovine rumen due to its microflora, polyunsaturated fatty acid content is very low in bovine meat [26]. A study conducted on calf, young bull, and cow meat confirmed this fatty acid development [27]. Animal species, breed, age, and farmed technique can affect fatty acids composition [28].

In view of the reduction of large amounts of by-products and wastes generated during meat processing, the aim of this study was to test the activity of eleven proteases in different reaction conditions for the hydrolysis of bovine fleshing meat and, after the selection of the four most efficient enzymes, to determine the molecular profile of the protein and lipid fractions of obtained hydrolysates. The peptide hydrolysates were also tested in vitro to assess their antioxidant and, for the first time, their anti-tyrosinase activities in view of their possible exploitation in the food and feed industrial sectors.

## 2. Materials and Methods

### 2.1. Reagents and Solvents

Deionized water was obtained from a Millipore Alpha Q-Waters purification system (Billerica, MA, USA). Sodium dihydrogen phosphate, hydrochloric acid, acetonitrile, DL-cystine, dichloromethane, N-acetyl-L-cysteine, sodium tetraborate decahydrate, DL-isoleucine and DL-norleucine, 30% hydrogen peroxide solution, sulfuric acid, formic acid, trifluoroacetic acid, 2′-azino-bis-3-ethylbenzothiazoline-6-sulfonic acid (ABTS), potassium persulfate, Trolox, tyrosinase from mushroom, and L-DOPA were purchased from Sigma-Aldrich (St. Louis, MO, USA). Copper oxide was obtained from Carlo Erba (Milan, Italy). O-phthalaldehyde and boric acid were bought from Fluka (Buchs, Switzerland). Sodium dodecyl sulfate was purchased from Biorad (Hercules, CA, USA). Kjeldahl tablets defoamers and catalyst 3.5 g/tablet were purchased from Merck (Darmstadt, Germany). AccQ-Fluor reagent kit was obtained from Waters (Milford, MA, USA).

### 2.2. Raw Material

Samples of calf fleshing were provided by Inalca Industria Alimentare Carni SpA (Castelvetro di Modena, Italy). The samples (three biological replicates of about 600 gFW each collected on a single slaughter day) were obtained from commercial breeds slaughtered in an abattoir at Inalca and transported to the University laboratories on the day of the slaughtering. The samples were grounded and homogenized in a kitchen blender (Kenwood FP735 1000 W, Tokyo, Japan) at 3 °C. The samples were divided in 5.0 ± 0.3 gFW (grams of fresh weight) aliquots and immediately frozen at −80 °C until further analyses.

### 2.3. Raw Material Chemical Composition

Moisture content of fleshing was determined using the official AOAC (Association of Official Analytical Chemists) method [29] according to Anzani et al. [12].

### 2.4. Proteases Digestion

Just before hydrolysis, 10 mL of 10 mM sodium-phosphate buffer (pH 7.5) were added to 5 gFW of fleshing; the final pH of the digestion mixture was 7.2 ± 0.2. Initially, the proteolytic activity of 11 different commercial proteases were initially tested following these conditions: 2% E/S ratio (g enzyme/gFW sample), 4 h of incubation time, pH 7.2–7.4 (with the exception of pepsin, which was tested at pH 3.0, in water acidified by chloridric acid), 140 rpm stirring, and specific temperature enzyme according to manufacturer’s instructions: Alcalase 1.5 MG (60 °C, Novozymes A/S, Denmark), protamex (60 °C, Novozymes A/S, Denmark), papain from *Carica papaya* (60 °C, Sigma-Aldrich, Milan, Italy), neutrase 1.5 MG (50 °C, Novozymes A/S, Denmark), flavourzyme 500 MG (50 °C, Novozymes A/S, Denmark), pancreatin from porcine pancreas (37 °C, Sigma-Aldrich, Milan, Italy), trypsin (type A, 13,000–20,000 U/mg, 37 °C, Sigma-Aldrich, Milan, Italy), trypsin from porcine pancreas (type B, 1000–2000 U/mg, 37 °C, Sigma-Aldrich, Milan, Italy), bromelain from pineapple stem (37 °C, Sigma-Aldrich, Milan, Italy), α-chymotrypsin from bovine pancreas (37 °C, Sigma-Aldrich, Milan, Italy), and pepsin from porcine gastric mucosa (37 °C, pH 3.0, Sigma-Aldrich, Milan, Italy). Control samples without any enzyme addition (ND, not digested) were performed by incubating the samples for 4 h, 140 rpm stirring, at each incubation temperature (ND 60 °C, 50 °C and 37 °C). The enzyme was inactivated by boiling the samples in a water bath for 5 min. The samples were cooled on ice and centrifuged at 5000 rpm for 15 min at 4 °C (Eppendorf centrifuge 5804 R, rotor A 4-44, Hamburg, Germany). After the centrifugation, three different fractions were observed: Pellet (residual solid material), intermediate liquid fraction (containing digested proteins/peptides/amino acids), upper fraction (containing lipids). The three fractions were separately collected and stored at −20 °C until following analyses.

Because of the gelatine formation in ND (60 °C, 4 h), α-chymotrypsin (60 °C, 4 h), and pepsin (37 °C, 4 h) samples, it was not possible to separate the three sub-fractions in these treatments, therefore they were discarded.

To optimize the E/S ratio and the time of incubation, several digestion treatments were performed for each type of enzyme. The four best digestion conditions were selected, yielding the highest amount of hydrolyzed proteins in the intermediate fraction measured as g of bovine serum albumin equivalents per liter (g BSA eq/L) [30]. The complete list of tested hydrolysis conditions and related yields is summarized in Supplementary Table S1.

### 2.5. Quantification of Free and Total Amino Acids, SDS-PAGE and Degree of Hydrolysis (DH) Analyses

The free amino acid determination in intermediate protein-based fractions, performed by high-performance liquid chromatography with fluorescence detection (HPLC/FLD) after AccQ•Tag derivatization, the SDS-PAGE (Sodium Dodecyl Sulphate - PolyAcrylamide Gel Electrophoresis) analysis, and the DH analysis by (o-phthaldialdehyde) OPA method, were performed as reported by Anzani et al. [12]. The DH was calculated as follows:

DH (%) = (N_free_/N_total_) × 100 (1)

where N_free_ represent the moles of free nitrogen atoms from α- amino groups of amino acids after hydrolysis measured by the OPA assay, and N_total_ represent the total moles of nitrogen atoms in solution before hydrolysis calculated by the ratio of total grams of proteins and the average residual amino acid molecular mass (MW 110 Da).

### 2.6. Peptide Identification by Linear Trap Quadrupole (LTQ)-Orbitrap Analyses

Peptide identification was performed by LTQ-Orbitrap analyses according to a method previously reported [12].

### 2.7. Fatty Acid Analysis

The fatty acid composition of upper fractions was determined as fatty acid methyl esters (FAMEs) by capillary gas chromatography analysis after alkaline treatment [31]. Methyl tridecanoate (C13:0, 2 mg/mL) was used as internal standard, and a GC 2010 Plus gas chromatograph (Shimadzu Corporation, Kyoto, Japan) equipped with a flame ionization detector (FID) and an AOC-20s auto sampler (Shimadzu Corporation) was used, according to Verardo et al. [32] with slight modifications. A BPX70 fused silica capillary column (10 m × 0.1 mm i.d. 0.2 µm film thickness; SGE Analytical Science, Ringwood, VIC. Australia) was used. The injector and flame ionization detector temperatures were set at 250 °C. Hydrogen was used as carrier gas at a flow rate of 0.8 mL/min. The oven temperature was held at 50 °C for 0.2 min, increased to 175 °C at 120 °C/min, held at 175 °C for 2 min, and finally increased from 175 to 220 °C at 20 °C/min. Samples were injected in split mode (0.3 µL) with a split ratio set at 1:100. Peak identification was accomplished by comparing peak retention time with GLC-463 standard mixture from Nu-Check (Elysian, MN, USA) and FAME 189-19 standard mixtures from Sigma-Aldrich Chemicals (St. Louis, MO, USA) and expressed as weight percentage of total FAMEs. FAMEs composition was measured in 2 replicates for each lipid extract (n = 6) and each analysis lasted 7 min.

### 2.8. Antioxidant and Anti-Tyrosinase Activity Evaluation

In all experiments, in vitro antioxidant activity of intermediate fractions was assessed by the 2′-azino-bis-3-ethylbenzothiazoline-6-sulfonic acid (ABTS) assay as reported by Ferri et al. [33]. The results were expressed as Trolox Equivalents Antioxidant Capacity (TEAC) by means of a dose–response calibration curve (between 0 and 2.5 µg of Trolox). Anti-tyrosinase capacity was measured by an optimized enzyme inhibition assay [24]. The brown color formation kinetic was detected (absorbance at 490 nm) in the presence of 10 U of tyrosinase, 2 mM L-DOPA (3,4-dihydroxy-L-phenylalanine), and the sample. The results were expressed as % of inhibition.

### 2.9. Statistical Analysis

All the treatments were performed in two biological replicates and two technical replicates were analyzed for each repetition. Statistically significant differences were analyzed among the same type of analytical data, related to different hydrolysate samples, by using one-way ANOVA followed by post hoc Tukey’s multiple pairwise comparison (*p* < 0.05) (R Software, version 1.3.5, R Core Tram, Vienna, Austria).

## 3. Results and Discussion

### 3.1. Calf Fleshing Bulk Characterization

Non-treated calf fleshing meat samples (ND) were analyzed. Proteins, fat, and moisture percentages found were 16% (± 4), 14% (± 2), and 72% (± 3), respectively, over the total wet weight. Previous data obtained on the same initial by-product [12] showed the presence of a lower fat content. This difference could be ascribable to calf samples’ biological variability, which could show a slightly different composition of the residual fleshing. Conversely, the distribution of total amino acids (Table 1) was similar to that reported by Anzani et al. [12], with Hyp, Glu, and Pro being the most abundant. In agreement with Rai et al. [34] and Bhaskar et al. [35], the presence of these amino acids suggested that fleshing is constituted by a great amount of collagen because of its proximity to the skin. A further important point of discussion is to evaluate the impact of the fleshing from a nutritional point of view. Therefore, the amount of essential amino acids was compared with that of a reference standard amino acid profile from raw lean beef [36]. Fleshing showed a lower amount of essential amino acids compared to the reference, demonstrating the lower nutritional value of fleshing compared to lean meat, given the presence of the collagen as fleshing main component. Collagen, as previously mentioned, is rich in Hyp, Pro, and Glu, and consequently caused a decrease of the essential amino acids in fleshing.

### 3.2. Optimization of the Enzymatic Reaction

**Figure 1:**
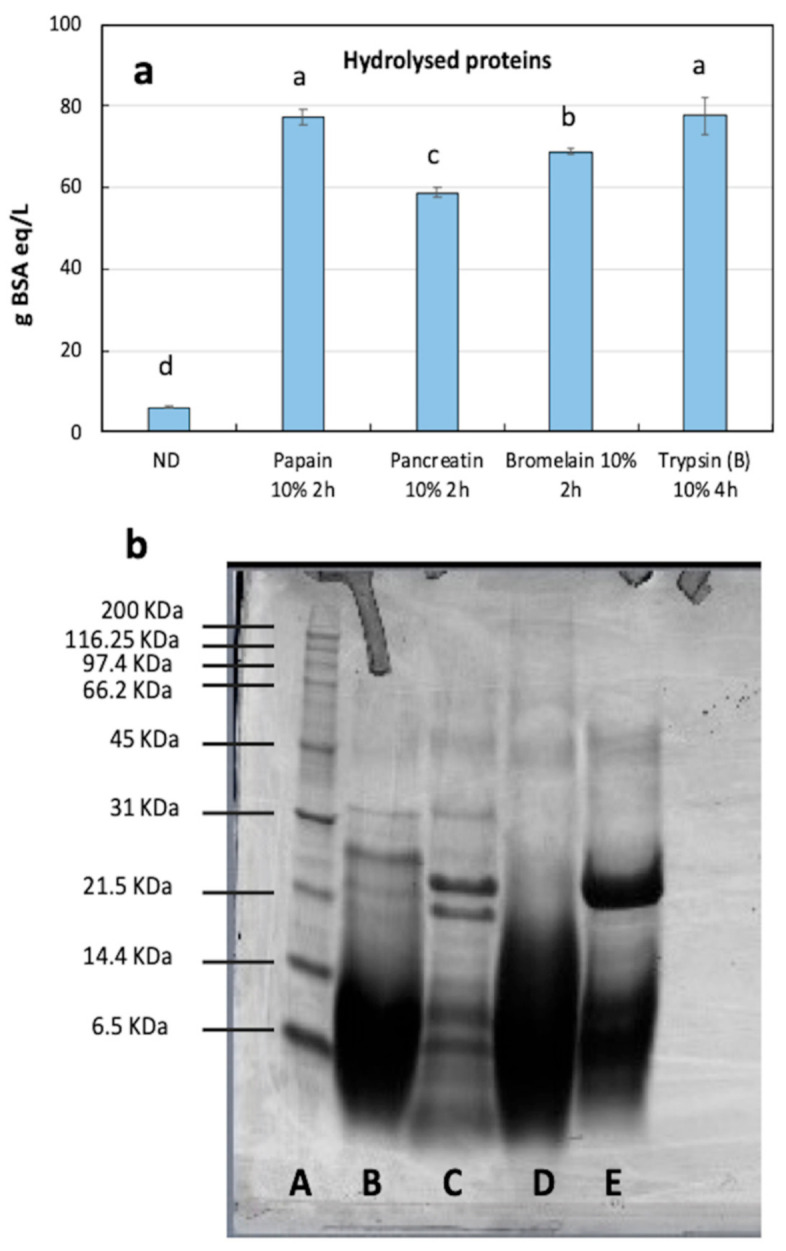
Optimized hydrolysis. (**a**) Quantification of hydrolyzed proteins (g BSA eq/L) in intermediate fractions obtained after calf fleshing hydrolysis at different digestion conditions. ND, average of not-digested control at 50 °C and 37 °C. Statistically significant differences among different hydrolysate samples were indicated by different letters, according to one-way ANOVA followed by post hoc Tukey’s multiple pairwise comparison (*p* < 0.05). Data are the mean ± SD (*n* = 4). (**b**) SDS-PAGE (Sodium Dodecyl Sulphate - PolyAcrylamide Gel Electrophoresis) profile of samples: A, standard marker; B, papain hydrolysate; C, pancreatin hydrolysate; D, bromelain hydrolysate; E, trypsin (B) hydrolysate.

In preliminary digestion trials, 11 different proteases were tested on calf fleshing samples. To assess the efficiency of the digestion reactions, the hydrolyzed protein amount as well as the antioxidant activity were measured, by spectrophotometrical assays, in the intermediate liquid protein-based fraction of hydrolysates (Supplementary Table S1). After preliminary trials, the four best digestion conditions, yielding the highest amount of digested proteins, were selected and were, respectively: papain: 10% E/S, 2 h 60 °C; pancreatin 10% E/S, 2 h 37 °C; bromelain 10% E/S, 2 h 37 °C; trypsin (type B): 10% E/S, 4 h 37 °C. Overall, the highest amount of hydrolyzed proteins was equally measured in samples obtained with 10% papain and 10% trypsin B (77.31 and 77.65 g BSA eq/L, respectively) followed by bromelain (68.88 g BSA eq/L) and pancreatin (58.76 g BSA eq/L) (Figure 1a, Supplementary Table S1).

The SDS-PAGE sample profile (Figure 1b) showed that the hydrolysates were constituted by peptides with a molecular weight below 31 kDa. However, there were some differences among the samples as consequence of the different cleavage sites of each enzyme, which could attack the sequence within specific amino acid residues or be unspecific. Of the selected enzymes, bromelain does not have a specific cleavage site while papain is a cysteine endopeptidase, hydrolyzing proteins, peptides, amides, and esters of amino acids and peptides, with the specific target of Arg, Lys, Glu, His, Gly, and Tyr bonds [37]. However, if the incubation is prolonged, further bonds are split. According to Olsen et al. [38], the cleavage site of trypsin is very specific cutting exclusively the C-terminal Arg or Lys. On the contrary, pancreatin cleavage site is not specific being a mixture of different type of enzymes such as protease, amylase, and lipase. However, the profile of the sample treated with pancreatin and trypsin type B resulted almost similar, with a blurred band lower than 14.4 kDa and 2 bands around 21.5 kDa (probably because of the high concentration in the sample treated with trypsin showed only one blurred band) (Figure 1b, lanes C and E).

### 3.3. Protein Fraction Analysis

The degree of hydrolysis (DH), which is directly linked to the enzyme proteolytic activity, was determined on selected samples on the basis of the number of the cleaved peptide bonds and on the amount of free amino acids present in solution [39]. DH was determined by monitoring the reaction of the free amino groups with OPA depending on the protein material released in solution. The sample treated with papain showed the highest DH (70% ± 2), followed by bromelain and pancreatin (55% ± 1 and 60% ± 5, respectively), which resulted to be not significantly different (*p* < 0.05). The sample obtained with trypsin B showed the lowest DH (25% ± 0.4) possibly due to the highest specificity of this enzyme, which is able to cleave the sequence after Arg and Lys and therefore release long peptides and less free amino acids. The present DH results were higher than literature data; for example, Zhang et al. [9] treated bovine collagen with six different proteases and diverse pre-treatments, obtaining DH always lower than 20%. The content and distribution of free amino acids were also measured and are reported in Table 2.

Particularly in the case of trypsin B and pancreatin digested samples, the most abundant amino acids were Arg (14.27 and 16.63%, respectively) and Lys (22.51 and 14.89%, respectively), according to the specificity of the cleavage site. The sample treated with papain was rich of Arg (14.58%), Lys (15.36%), Leu (11.20%), and Gly (9.00%), while that obtained with bromelain showed high levels of Ile (19.26%), Pro (11.89%), Lys (10.55%), Leu (9.04%), and Thr (8.37%).

Moreover, the hydrolysates were also analyzed by high-resolution mass spectrometry to identify the most abundant peptides present in the origin proteins (Table 3). In the pancreatin and papain hydrolysates most of the detected peptides derived from collagen. On the other side, in trypsin B-derived digestates the most abundant peptides were originated from muscular protein, such as myosin.

### 3.4. Antioxidant and Anti-Tyrosinase Activity

**Figure 2:**
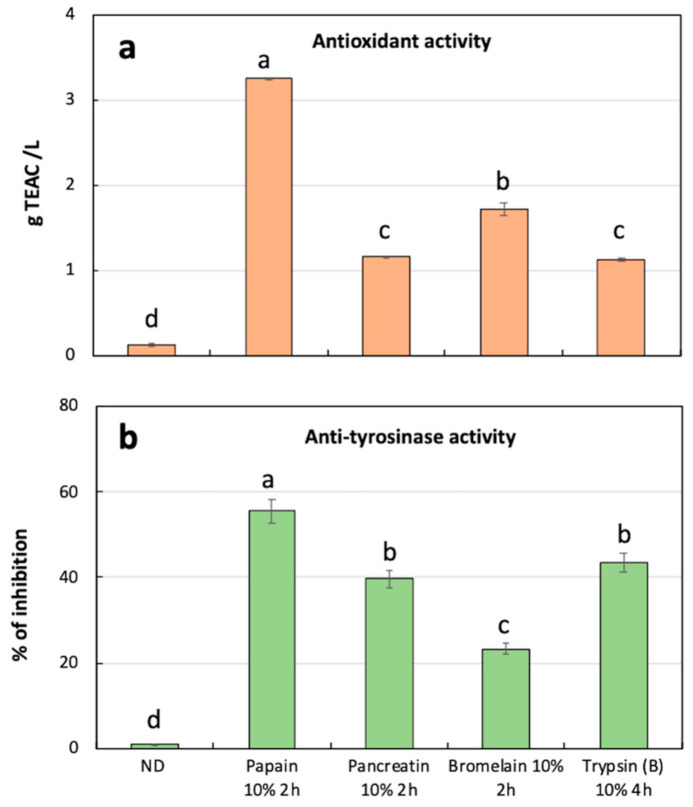
Quantification of antioxidant activity (**a**) and anti-tyrosinase activity (**b**) in intermediate fractions obtained after calf fleshing hydrolyses at different digestion conditions. ND, average of not digested control at 50 °C and 37 °C. Statistically significant differences among the same analysis were indicated by different letters, according to one-way ANOVA followed by post hoc Tukey’s multiple pairwise comparison (*p* < 0.05). TEAC, Trolox equivalents antioxidant capacity. Data are the mean ± SD (*n* = 4).

The antioxidant activity was assayed in intermediate fraction of all the samples (Supplementary Table S1) and the highest values were obtained using 10% papain (on average 3.22 g TEAC/L) (Supplementary Table S1, Figure 2a). These results are comparable with literature data by Ryder et al. [40] who obtained hydrolysates, from bovine connective tissue treated with different fungal proteases, showing an average Trolox equivalence lower than 10 mmol/L (equals to 2.5 g TEAC/L), increased to 45 mmol/L (equals to 11 g TEAC/L) by a more complex proteolytic protease preparation.

In addition, the papain-sample (10% for 2 h) also showed the highest inhibition activity (55.6%) of tyrosinase enzyme, while the sample digested with bromelain (10% for 2 h) showed the lowest (23.6%) (Figure 2b). To the best of the authors’ knowledge, this is the first report on anti-tyrosinase activities of fleshing meat hydrolysates. A comparison can be done with tyrosinase inhibitory activity of a fish meat (*Scomber japonicus*) hydrolysed by subcritical water treatment, which shows best activities between 48% and 65.5% of inhibition depending on process conditions [41]. These results were, therefore, very promising, as nowadays, the interest in anti-tyrosinase ingredients is increasing among several industries [19,20]. Fleshing meat hydrolysates exerting this type of bioactivity can be exploited as browning-preventing agents already in the meat-processing chain, for example as preservative ingredients in food preparations, or as additives for the production of food active packaging.

### 3.5. Fatty Acid Composition

A total of 17 individual fatty acids were identified and quantified in the upper fraction of differently hydrolyzed samples. As shown in Table 4, oleic acid (C18:1 cis9) was the most abundant fatty acid in all digestates, ranging from about 39.59 to 42.80%. The second major fatty acid detected was palmitic acid (C16:0, 24–26%), followed by stearic acid (C18:0, 7–9%), palmitoleic acid (C16:1 cis, 5–6%), myristic acid (C14:0, 5–6%), and linoleic acid (C18:2 n6, 4–5%). The samples digested with four different enzymes differed for the fatty acid composition but not for the concentration of them, that did not show significant differences (*p* < 0.05).

As shown in Table 4, the sample digested with papain showed the most complete fatty acids composition, which was the same of sample digested with trypsin, except for C18:1 t9 that was absent. Samples treated with pancreatin and bromelain showed a more limited fatty acids composition. In particular, C12:0, C15:0, C16:1t, C17:0, C17:1, C18:1t9, C18:3n3, and C20:0 were absent in these two samples; C20:3n6 was present only in the sample digested with pancreatin. Saturated fatty acids (SFA) were present in considerable amounts, from 39.52 to 40.46%, whereas mono-unsaturated fatty acids (MUFA) were the class more abundant in all the samples ranging from 50.20 to 54.60% without significant differences (*p* < 0.05) for both classes among the different samples. Polyunsaturated fatty acids (PUFA), instead, showed a significantly higher amount in the sample digested with pancreatin (10.03%) and in a range from 4.99 to 6.39% in the other three samples. The low PUFA content of the analyzed calf’s by-products is related to animal nature, and rumen microflora in bovine rumen is responsible for the hydrogenation process, which takes place at unsaturated fatty acids, increasing the total SFA amount [26,27]. Compared to the literature [42,43,44], the results obtained in this work revealed lower amount of saturated and polyunsaturated fatty acids and higher concentration of monounsaturated fatty acids. In fact, in studies conducted by Baggio et al. [42], Muchenje et al. [43], and Brugiapaglia et al. [44], SFA concentrations were, respectively, 47–48%, 44–45% and 46–49%; MUFA concentrations were 39–40%, 34–36%, and 32–42%; and PUFA concentrations were 12–14%, 19–22%, and 41–22%.

## 4. Conclusions

In the present paper, the best four enzyme protocols for the hydrolysis of calf fleshing meat were selected and optimized. These were: Papain (10% E/S, for 2 h at 60 °C); pancreatin (10% E/S, for 2 h at 37 °C); bromelain (10% E/S, for 2 h at 37 °C); and trypsin (type B) (10% E/S, for 4 h at 37 °C). SDS-PAGE profile, free-amino acid contents, DH, and peptide identification results pointed out that the differences between the hydrolysates were due to the specificity of the enzyme’s cleavage site. In the case of the fatty acid composition, MUFA were the most abundant ranging from 50.20 to 54.60%; whereas the digestate obtained with pancreatin showed the highest amount of PUFA (10.03%). The obtained protein-based samples also showed both antioxidant and anti-tyrosinase biological activities with papain-digested samples displaying the highest bioactivities (up to 3.52 g TEAC/L antioxidant capacity and to 55.6% tyrosinase inhibition). These results demonstrated the potential use of the enzymatic hydrolysis as a way of recovering calf fleshing meat by the production of hydrolysates reach in proteins, peptides, and free amino acids with anti-oxidant and anti-tyrosinase activities, and of good quality fatty acids fractions like n-3 and n-6 series. As no solvents nor chemicals were used along the exploitation process, the present results suggest that a possible future valorization of isolated fractions from calf fleshing by-products, as food or feed ingredients, is feasible and could be implemented short term within the meat-processing pipeline.

## Figures and Tables

**Table 1:** High-performance liquid chromatography with fluorescence detection (HPLC-FLD) analysis of amino acid distribution in initial calf fleshing by-products given as percentage of the total amino acid pool.

Amino Acids	% on Total Amino Acids	Reference (%)
Hyp	12.1 ± 0.6	1.4
Asp	7.0 ± 0.5	9.4
Ser	3.2 ± 0.1	4.2
Glu	13.2 ± 0.7	15.6
Gly	8.2 ± 0.6	7.1
His	**2.2 ± 0.3**	**3.4**
Arg	6.3 ± 0.5	6.8
Thr	**3.4 ± 0.3**	**4.0**
Ala	5.3 ± 0.8	6.5
Pro	10.7 ± 1.2	5.3
Tyr	2.7 ± 0.4	3.2
Val	**3.3 ± 0.2**	**5.1**
Met	**3.4 ± 0.3**	**2.7**
Lys	**6.6 ± 0.4**	**8.6**
Ile	**3.3 ± 0.3**	**4.6**
Leu	**5.8 ± 0.5**	**8.1**
Phe	**3.4 ± 0.3**	**4.1**

Data in bold show the amounts of essential amino acids. The reference is represented by standard amino acid profile from raw lean beef [36]. Data (means ± SD) are expressed in (%).

**Table 2:** Free amino acid distribution of the hydrolysates obtained by enzymatic hydrolysis of calf fleshing meat.

Amino Acids	Papain	Pancreatin	Bromelain	Trypsin (B)
**Hyp**	3.46 ± 0.33 ^b^	3.54 ± 0.07 ^b^	2.79 ± 0.60 ^b^	5.75 ± 0.30 ^a^
**Asp**	0.14 ± 0.03 ^c^	1.81 ± 0.01 ^a^	0.81 ± 0.01 ^b^	1.78 ± 0.36 ^a^
**Ser**	3.55 ± 0.06 ^a^	4.17 ± 0.66 ^a^	3.73 ± 0.20 ^a^	1.75 ± 0.01 ^b^
**Glu**	4.66 ± 0.04 ^b^	6.86 ± 0.25 ^a^	2.44 ± 0.37 ^c^	2.77 ± 0.05 ^c^
**Gly**	9.00 ± 0.02 ^a^	1.67 ± 0.15 ^c^	3.27 ± 0.13 ^b^	1.57 ± 0.04 ^c^
**His**	2.62 ± 0.06 ^c^	5.80 ± 0.08 ^a^	4.65 ± 0.16 ^b^	2.47 ± 0.02 ^c^
**Arg**	14.58 ± 0.29 ^a^	16.63 ± 1.69 ^a^	3.74 ± 0.34 ^b^	14.27 ± 1.04 ^a^
**Thr**	2.93 ± 0.08 ^b^	3.79 ± 0.07 ^b^	8.37 ± 0.24 ^a^	3.48 ± 0.05 ^b^
**Ala**	3.44 ± 0.03 ^b^	3.70 ± 0.67 ^a^	2.29 ± 0.09 ^b^	3.28 ± 0.80 ^b^
**Pro**	7.67 ± 0.04 ^b^	1.32 ± 0.23 ^c^	11.89 ± 0.62 ^a^	1.36 ± 0.21 ^c^
**Tyr**	7.89 ± 0.21 ^a^	8.93 ± 0.86 ^a^	4.96 ± 0.47 ^b^	7.52 ± 0.73 ^a^
**Val**	1.85 ± 0.09 ^c^	4.43 ± 0.15 ^a^	3.08 ± 0.01 ^b^	3.46 ± 0.69 ^b^
**Met**	3.93 ± 0.13 ^b^	4.20 ± 0.41 ^b^	4.55 ± 0.22 ^b^	5.69 ± 0.03 ^a^
**Lys**	15.36 ± 0.13 ^b^	14.89 ± 1.84 ^b^	10.55 ± 0.06 ^c^	22.51 ± 1.89 ^a^
**Ile**	3.96 ± 0.12 ^b^	4.05 ± 0.20 ^b^	19.26 ± 0.23 ^a^	4.47 ± 0.69 ^b^
**Leu**	11.20 ± 0.47 ^a^	7.82 ± 0.33 ^c^	9.04 ± 0.16 ^b^	9.45 ± 0.38 ^b^
**Phe**	3.75 ± 0.33 ^c^	6.40 ± 0.58 ^b^	4.59 ± 0.09 ^c^	8.40 ± 0.45 ^a^

Data (means ± SD) are expressed in (%). Different lowercase letters on the same row show significantly different values for samples digested with different enzyme determined one-way ANOVA followed by post hoc Tukey’s multiple pairwise comparison (*p* < 0.05).

**Table 3:** Identification by Linear Trap Quadrupole-OrbiTrap of the main peptides in digestates obtained after enzymatic hydrolysis of calf fleshing meat, and of the proteins of origin. Proteins are listed according to the highest number of peptides generated [12]. AA, amino acids.

Enzyme	Identified Peptides	Parent Protein	Average Peptide Length (N°AA)
**Papain**	78	Collagen alpha-1(I) chain	14
	71	Actin, alpha skeletal muscle	11
	56	Collagen alpha-2(I) chain	13
	54	Collagen alpha-1(II) chain	12
	30	Myosin-1	11
**Pancreatin**	70	Titin	12
	57	Collagen alpha-1(I) chain	11
	53	Collagen alpha-2(I) chain	10
	29	Collagen, type III, alpha 1	14
	24	IgG heavy chain	18
**Bromelain**	24	Collagen alpha-1(I)	9
	17	Hemoglobin subunit beta	23
	15	Hemoglobin subunit alpha	25
	15	Collagen alpha-2(I) chain	9
	13	Myosin light chain 1/3, skeletal muscle isoform	13
**Trypsin (B)**	59	Titin	11
	46	Myosin-2	11
	28	Myosin-1	10
	23	Collagen alpha-1(I) chain	13
	10	Myosin-7	12

**Table 4:** Fatty acids (FA) identification and quantification of the hydrolysates obtained by enzymatic hydrolysis of fleshing meat.

FA	Papain	Pancreatin	Bromelain	Trypsin (B)
**C12:0**	0.69 ± 0.00	n.d.	n.d	0.51 ± 0.02
**C14:0**	5.82 ± 0.09	5.88 ± 0.04	5.66 ± 0.30	5.41 ± 0.33
**C14:1c**	2.30 ± 0.19	2.67 ± 0.56	3.79 ± 0.15	2.14 ± 0.01
**C15:0**	0.31 ± 0.01	n.d	n.d	0.28 ± 0.00
**C16:0**	24.66 ± 0.03	23.99 ± 1.95	26.55 ± 0.38	24.72 ± 0.19
**C16:1 t**	0.37 ± 0.02	n.d	n.d	0.27 ± 0.07
**C16:1 c**	5.80 ± 0.02	5.89 ± 0.60	5.88 ± 0.96	5.92 ± 0.28
**C17:0**	0.58 ± 0.01	n.d	n.d	0.59 ± 0.07
**C17:1**	0,61 ± 0.04	n.d	n.d	0.64 ± 0.10
**C18:0**	7.77 ± 0.03	9.91 ± 0.66	8.21 ± 0.47	7.59 ± 0.40
**C18:1 t9**	1.56 ± 0.11	n.d	n.d	n.d
**C18:1 c9**	40.52 ± 0.02	39.59 ± 4.69	42.80 ± 1.97	41.61 ± 1.37
**C18:1 c11**	1.99 ± 0.14	2.06 ± 0.42	2.12 ± 0.32	2.13 ± 0.10
**C18:2 n6**	4.91 ± 0.15	4.78 ± 0.38	4.99 ± 0.95	4.38 ± 0.60
**C18:3 n3**	0.71 ± 0.08	n.d	n.d	0.90 ± 0.08
**C20:0**	0.64 ± 0.06 ^a^	n.d	n.d	0.42 ± 0.01 ^b^
**C20:3 n6**	0.76 ± 0.66	5.24 ± 0.77	n.d	0.85 ± 0.10
**SFA**	40.46 ± 0.08	39.77 ± 0.75	40.41 ± 1.16	39.52 ± 0.98
**MUFA**	53.16 ± 0.51	50.20 ± 3.15	54.60 ± 2.11	54.36 ± 1.40
**PUFA**	6.39 ± 0.59 ^b^	10.03 ± 2.39 ^a^	4.99 ± 0.95 ^c^	6.12 ± 0.42 ^b^

SFA, saturated fatty acid; MUFA, monounsaturated fatty acid; PUFA, polyunsaturated fatty acid. n.d., not determined. Data (means ± SD) are expressed in mg FA/100 mg fatty acid methyl esters (FAME) (%). Different letters on the same row show significantly different values for samples digested with different enzyme determined by one-way ANOVA followed by post hoc Tukey’s multiple pairwise comparison (*p* < 0.05).

## Data Availability

All data are available within the manuscript and Supplmentary Material.

## Supplementary Materials

The following are available online at https://www.mdpi.com/article/10.3390/foods10040755/s1, Table S1: Preliminary optimisation of calf fleshing meat digestion conditions and selection of most efficient proteases.
