# Supplementary material for: Enzymatic Digestion of Calf Fleshing Meat By-Products: Antioxidant and Anti-Tyrosinase Activity of Protein Hydrolysates, and Identification of Fatty Acids
Source: Foods. 2021 Apr 2;10(4):755. doi: 10.3390/foods10040755 (PMC8067011; doi:10.3390/foods10040755)
Supplement: Supplementary file 1 [file foods-10-00755-s001.pdf]

Supplementary Table S1. Preliminary optimisation of calf fleshings meat digestion conditions and selection of most efficient proteases. Content of proteins (expressed as g of bovine serum albumin equivalent per L, g BSA eq/L) and antioxidant activity (expressed as g of Trolox equivalent per L, g TEAC /L) on digested intermediate protein-based fractions. ND, not digested samples. Samples ND 60°C (4h), α-Chymotrypsin and Pepsin (2%, 4h) showed the formation of gelatin after incubation and it was not possible to separate their intermediate liquid fraction and perform the assays.N.d., not determined. In bold samples selected for the further assays.

**Enzyme**	**Digestion conditions**			**Hydrolysed proteins (g BSA eq/L)**	**Antioxidant activity (g TEAC /L)**
	**E/S ratio (%) (g enzyme/gFW sample)**	**Incubation time (h)**	**Temperature (°C)**		
**ND**	-	4	60	n.d.	n.d.
	-	4	50	3.34 ± 0.12	0.14 ± 0.04
	-	4	37	8.52 ± 0.34	0.11 ± 0.01
**Alcalase**	1	2	60	50.55 ± 3.17	0.78 ± 0.02
	2	2	60	49.57 ± 1.15	2.27 ± 0.03
	2	4	60	53.17 ± 0.06	2.29 ± 0.01
	2	6	60	51.50 ± 1.70	2.20 ± 0.02
	2	24	60	56.37 ± 2.89	2.28 ± 0.02
	4	2	60	56.05 ± 4.93	0.90 ± 0.19
	10	4	60	55.10 ± 4.72	1.37 ± 0.02
	10	24	60	46.44 ± 1.20	1.94 ± 0.15
**Protamex**	1	2	60	34.93 ± 0.59	0.49 ± 0.03
	2	2	60	47.37 ± 2.91	2.11 ± 0.04
	2	4	60	49.63 ± 2.21	2.29 ± 0.02
	2	6	60	48.21 ± 5.32	2.14 ± 0.12
	2	24	60	29.44 ± 1.34	1.90 ± 0.04
	4	2	60	45.71 ± 2.74	0.97 ± 0.05
	10	4	60	49.26 ± 1.58	2.22 ± 0.25
	10	24	60	31.70 ± 2.30	2.05 ± 0.01
**Papain**	2	2	60	56.35 ± 2.76	0.90 ± 0.14
	2	4	60	55.83 ± 0.79	2.11 ± 0.04
	2	24	60	42.14 ± 5.00	2.06 ± 0.10

	5	2	60	59.03 ± 1.49	1.25 ± 0.23
	8	2	60	68.75 ± 1.32	1.56 ± 0.13
	10	2	60	**77.31 ± 1.85**	3.25 ± 0.01
	10	4	60	73.32 ± 10.13	3.12 ± 0.04
	10	6	60	75.92 ± 0.49	3.09 ± 0.08
	10	24	60	69.54 ± 3.19	3.42 ± 0.04
**Neutrase**	2	4	50	39.17 ± 3.15	1.43 ± 0.04
	2	24	50	33.32 ± 0.95	2.39 ± 0.01
	10	4	50	40.62 ± 4.19	1.94 ± 0.01
	10	24	50	35.62 ± 1.34	2.54 ± 0.04
**Flavourzyme**	2	4	50	24.51 ± 2.29	0.96 ± 0.18
	2	24	50	19.08 ± 1.70	2.30 ± 0.07
	10	4	50	22.83 ± 1.04	1.36 ± 0.13
	10	24	50	16.14 ± 2.35	1.86 ± 0.08
**Pancreatin**	1	4	37	40.57 ± 1.58	0.59 ± 0.07
	2	4	37	47.63 ± 0.10	0.60 ± 0.01
	5	4	37	46.75 ± 1.47	0.70 ± 0.06
	8	4	37	55.45 ± 0.93	0.86 ± 0.08
	10	2	37	**58.76 ± 1.98**	1.16 ± 0.01
	10	4	37	58.16 ± 2.16	0.98 ± 0.11
**Trypsin (type A)**	1	4	37	36.54 ± 1.81	0.44 ± 0.04
	2	4	37	33.45 ± 1.94	0.44 ± 0.02
	4	4	37	60.09 ± 1.97	1.03 ± 0.15
	6	4	37	61.76 ± 0.86	0.82 ± 0.05
**Trypsin (type B)**	6	4	37	64.12 ± 3.12	1.04 ± 0.02
	10	2	37	70.40 ± 1.29	1.01 ± 0.03
	10	4	37	**77.65 ± 4.53**	1.12 ± 0.02
**Bromelain**	1	4	37	48.42 ± 3.95	0.66 ± 0.06
	2	4	37	49.82 ± 2.23	0.89 ± 0.01
	5	4	37	56.48 ± 4.45	1.09 ± 0.15

	8	4	37	65.53 ± 3.69	1.70 ± 0.03
	10	2	37	**68.88 ± 0.57**	1.72 ± 0.07
	10	4	37	68.16 ± 0.13	1.73 ± 0.04
**α-Chymotrypsin**	2	4	37	n.d.	n.d.
**Pepsin**	2	4	37	n.d.	n.d.
